# Reshaping of Gait Coordination by Robotic Intervention in Myelopathy Patients After Surgery

**DOI:** 10.3389/fnins.2018.00099

**Published:** 2018-03-02

**Authors:** Sandra Puentes, Hideki Kadone, Shigeki Kubota, Tetsuya Abe, Yukiyo Shimizu, Aiki Marushima, Yoshiyuki Sankai, Masashi Yamazaki, Kenji Suzuki

**Affiliations:** ^1^Faculty of Engineering, Information and Systems, University of Tsukuba, Ibaraki, Japan; ^2^Center for Innovative Medicine and Engineering, University of Tsukuba Hospital, Ibaraki, Japan; ^3^Department of Orthopaedic Surgery, Faculty of Medicine, University of Tsukuba Hospital, Ibaraki, Japan; ^4^Department of Rehabilitation Medicine, Faculty of Medicine, University of Tsukuba Hospital, Ibaraki, Japan; ^5^Department of Neurosurgery, Faculty of Medicine, University of Tsukuba Hospital, Ibaraki, Japan; ^6^Center for Cybernics Research, University of Tsukuba, Ibaraki, Japan

**Keywords:** myelopathy, motor deficit, gait coordination, kinematics, robotic therapy, gait reshaping

## Abstract

The Ossification of the Posterior Longitudinal Ligament (OPLL) is an idiopathic degenerative spinal disease which may cause motor deficit. For patients presenting myelopathy or severe stenosis, surgical decompression is the treatment of choice; however, despite adequate decompression residual motor impairment is found in some cases. After surgery, there is no therapeutic approach available for this population. The Hybrid Assistive Limb® (HAL) robot suit is a unique powered exoskeleton designed to predict, support, and enhance the lower extremities performance of patients using their own bioelectric signals. This approach has been used for spinal cord injury and stroke patients where the walking performance improved. However, there is no available data about gait kinematics evaluation after HAL therapy. Here we analyze the effect of HAL therapy in OPLL patients in acute and chronic stages after decompression surgery. We found that HAL therapy improved the walking performance for both groups. Interestingly, kinematics evaluation by the analysis of the elevation angles of the thigh, shank, and foot by using a principal component analysis showed that planar covariation, plane orientation, and movement range evaluation improved for acute patients suggesting an improvement in gait coordination. Being the first study performing kinematics analysis after HAL therapy, our results suggest that HAL improved the gait coordination of acute patients by supporting the relearning process and therefore reshaping their gait pattern.

## Introduction

The Ossification of the Posterior Longitudinal Ligament (OPLL) is an uncommon disease characterized by a pathological ectopic ligament ossification affecting usually the cervical or thoracic spine segments (Epstein, 2002; Kalb et al., 2011). This degenerative disease has high prevalence in Asian populations, with a high incidence in the Japanese community (Epstein, 2002; Kalb et al., 2011; Smith et al., 2011; Kommu et al., 2014); however, in the past years features of OPLL has been recognized also in patients from Europe and North America (Kalb et al., 2011). The disease onset typically affect patients in their fifties, and has an incidence twice as high for males than for females (Epstein, 1992; Choi et al., 2011; Kommu et al., 2014). Surgical decompression of the spinal cord is usually necessary when the medullar compression leads to symptomatic neurological deterioration (Mehdi et al., 2016); nonetheless, despite appropriate decompression residual motor impairment is found in some patients. The degree of canal stenosis, intramedullary conditions, initial motor score and patient age have been related with the outcome where the prognosis is less optimistic for older patients with severe neurologic deficit and marked myelopathy signs (Gu et al., 2015; Kwon et al., 2015). After decompression surgery, if gait disturbances are sustained there is no available intervention to support the motor rehabilitation of such patients.

The Hybrid Assistive Limb®(HAL) robot suit is a wearable powered exoskeleton designed to assist the voluntary control of hip and knee joint motion (Kawamoto et al., 2014). It has six degrees of freedom related to the sagittal movement of the bilateral hip, knee, and ankle joints. It has four motors distributed bilaterally, located over the lateral aspects of the hip and knee joints. The motors receive information from surface electrodes which are able to collect bioelectric signals from the action potential reaching the muscles during movement preparation and initiation. Such information is processed and used by the exoskeleton to assist the patient during gait training. In previous studies where HAL was used in neurologic patients with gait disturbances, improvement in clinical scores, and walking performance, such as elongation of the stride length and increment of gait speed, was found (Kawamoto et al., 2013; Sakakima et al., 2013; Fujii et al., 2016; Kubota et al., 2016; Sczesny-Kaiser et al., 2017). A group of stroke patients treated with HAL improved their sit-to-stand movements thanks to the increment of the forward tilt angle (Kasai and Takeda, 2016). Additionally, spinal cord injury patients showed improvement in their spasticity (Ikumi et al., 2016), recovery of lower limb muscle activities (Shimizu et al., 2017), reduction of neuropathic pain (Cruciger et al., 2016) and normalization of cortical excitability and cortical plasticity (Sczesny-Kaiser et al., 2017). However, there is no available data about gait kinematics evaluation after HAL therapy.

For gait kinematic analysis, joint angle measurements were used in the past; however, the pattern of the angles of flexion-extension at the hip and ankle joints tends to differ from each subject and vary largely depending on the gait speed (Borghese et al., 1996). On the other hand, the evaluation of the lower limbs with respect to the vertical when divided in three segments has shown a stereotyped pattern across different subjects without regard to gait pattern, speed, or anatomic discrepancies (Borghese et al., 1996). Described by Borghese et al. (1996) the so-called law of intersegmental coordination is a kinematic law that describes the coordination patterns given by the correlation of the elevation angles of the lower limb divided in three segments (thigh, shank, and foot) with respect to the vertical. From this point of view, the degrees of freedom of the lower limb are reduced to 3, and when the angles are plotted against each other, they covary presenting regular loops on a plane. Intersegmental coordination analysis has been used in conditions affecting gait pattern and coordination as stroke (Chow and Stokic, 2015) and cerebellar ataxia (Martino et al., 2014); and also for assessment after therapeutic interventions as botulin toxin injections for spasticity control (Bleyenheuft et al., 2009), ankle foot orthoses in stroke patients (Bleyenheuft et al., 2013), and combined therapy for Parkinson patients (Grasso et al., 1999). However, there is no report of planar covariation analysis in patients with gait impairment receiving robotic assisted therapy.

In this study, HAL therapy was applied to OPLL patients in an acute or chronic stage after decompression surgery. Clinical scores, walking performance, and kinematics analysis was performed before the first and after the last HAL therapy session. To our knowledge, this is the first study applying kinematic analysis in OPLL patients after HAL therapy.

## Materials and methods

### Patients

Twelve patients with a diagnosis of OPLL associated to severe motor symptoms and followed by decompression surgery joined the present study. The patients were distributed in acute (3 women, 2 men, age ± 59.6 years; starting at ± 24.4 days after surgery), and chronic groups (7 men, age ± 70.1 years; starting at ± 1151.4 days after surgery) accordingly to the time interval elapsed between decompression surgery and HAL therapy (Table 1). All the patients included in this study were able to voluntarily control their lower limbs. For acute patients, weight support was provided if necessary, so that the patient could produce gait by him/herself; all chronic patients were able to walk independently with or without cane support. Kinematics data from eight healthy volunteers (5 women, 3 men, age ± 57 years) who did not receive HAL treatment was also used for comparisons. This study was approved by the University of Tsukuba Hospital Ethics Committee (Approval number: H26-22) and implemented according to the ethical principles of the Declaration of Helsinki and the University Guidelines for Clinical Trials. All patients received a personalized explanation of the research contents, participation and data usage before signing an informed consent.

**Table 1:** Subjects characteristics.

**Participant ID**	**Group**	**Sex**	**Age**	**Surgery-HAL interval (days)**
A1	Acute	F	78	15
A2	Acute	M	64	26
A3	Acute	M	52	18
A4	Acute	F	63	32
A5	Acute	F	41	31
C1	Chronic	M	70	288
C2	Chronic	M	75	287
C3	Chronic	M	68	3,655
C4	Chronic	M	78	372
C5	Chronic	M	76	2,188
C6	Chronic	M	58	540
C7	Chronic	M	66	730
H1	Healthy	F	56	–
H2	Healthy	F	42	–
H3	Healthy	F	59	–
H4	Healthy	F	67	–
H5	Healthy	F	60	–
H6	Healthy	M	50	–
H7	Healthy	M	45	–
H8	Healthy	M	77	–

*Surgery-HAL interval refers to the days elapsed from surgery to the beginning of HAL therapy*.

### HAL setup

For this study, double leg version of robot suit HAL was used. Surface electrodes were placed to detect neuromuscular activity of Iliopsoas (hip flexor), Gluteus Maximus (hip extensor), Biceps Femoris (knee flexor), and Quadriceps (vastus lateralis, knee extensor); these signals were used by the robot to support a patients' gait. Four motors were placed bilaterally beside the patient's hip and knee (two per leg). A hip motor was actuated to produce torque in proportion to a weighted difference of filtered activation of flexor and extensor muscles of the hip. In the same manner, a knee motor was actuated using filtered activation of the knee's flexor and extensor muscles. The weights multiplied on the activation of each of the antagonistic pair of the muscles, and the overall gain, were adjusted manually for each patient's comfort through the HAL therapy sessions.

### Evaluation

Before starting the first HAL therapy session and after the last one, a functional evaluation was performed by using the modified Ranking Scale (mRS), Barthel Index (BI), Functional Independence Measure (FIM, motor score only), and the American Spinal Injury Association impairment scale (ASIA, motor score only) scores in order to evaluate the degree of dependence on daily life activities. Following, the patients were evaluated without fitting the robot by using the 10 m walk test where time and number of steps were counted while the patient walked 10 m in a straight line at a comfortable pace; the speed and stride length were calculated from this data.

### HAL therapy

The designed intervention consisted of 10 sessions of HAL therapy performed within the hospitalization period for acute patients. Chronic patients attended to the therapy in the hospital as outpatients. Sessions were carried out twice per week, 1 h per session divided in fitting, therapy, and releasing. Each session started by fitting HAL to the patient. A walking device (All-in-One Walking Trainer, Ropox A/S, Naestved, Denmark) with a harness was used to prevent falls, and to support body weight in some patients who needed it to walk. A HAL therapy session included 20 min of walking activity at a comfortable pace on a 25 m oval course with rest intervals. At the end of the session, the patient was released from the harness and robot. Vital signs including blood pressure, heart rate, and oxygen saturation were monitored at the beginning, end and within therapy intervals to ensure that patients were stable.

### Data collection and analysis

Before the initial and after the final session of HAL therapy, a motion capture system (VICON MX, 16 T20s cameras, 100 Hz) was used with Plug-in gait lower limbs marker-set to record segmental kinematics. Following the method described by Borghese et al. (1996), the lower limbs of the participants were analyzed regarding the elevation angles (EA) composed by the orientation of the limb segments in the sagittal plane with respect to the vertical. The evaluated segments (**Figure 2A**) were thigh (trochanter to lateral epicondyle of the femur), shank (lateral epicondyle of the femur to lateral malleolus), and foot (posterior calcaneal tuberosity to second metatarsal). For each leg of each participant, planar covariation of the EA was calculated by using a principal component (PC) analysis, after normalization by subtracting the mean value. In normal conditions, the first (PC1) and second (PC2) components covariates over a plane describing the shape of the gait loop; we assessed the proportional width of the covariance loop by the percentage of variance (PV) of the PC2 (PV2) (Martino et al., 2014). The third component (PC3) is orthogonal to the plane, showing the data component that deviates from the covariance plane. The PV represented by the PC3 (PV3) becomes an index of planarity of the loop, where 0% corresponds to an ideal plane, evaluating the proportional deviation from the covariance plane. Standard deviation of PC2 and PC3 scores (PC2-SD and PC3-SD) were computed to assess respectively the actual width of the covariance loop and the amount deviation from the covariance plane. To compare the plane orientation before and after HAL therapy, the unit vector U3 which is normal to the covariance plane was obtained as the third eigenvector of the covariance matrix. U3 is composed of the direction cosines of the normal vector (NV) against the coordinates of the thigh, shank, and foot. NV difference between planes before and after HAL therapy was calculated for acute and chronic groups. Following, to evaluate deviation of U3 from that of the healthy group, cosine deviation was calculated by the inner product of each patient's U3 against the averaged direction cosines (U3) of healthy volunteers group. Arccosine was calculated on the result of the inner product to obtain the angular deviation of U3 vector from averaged healthy patients.

In order to evaluate changes in the movement of EA range during gait, peak comparisons for each EA were made for acute and chronic groups. Gait cycles were extracted from the original data according to the movement of the toe and heel markers. Time variable was discarded to normalize the data from 0 to 100% to represent progression of gait for each cycle, and then averaged cycle profile was obtained for each elevation angle for each subject. Comparisons from the highest and lowest peaks and their difference were calculated for each EA. Data was plotted for acute and chronic groups and data from healthy volunteers was plotted along as reference.

Following, the distance from each data point to the covariance plane was calculated and comparisons were made before and after HAL therapy for each group. To graphically present the pattern of deviation from the covariance plane through gait cycle, a Kernel method (Kim and Scott, 2012) was used to create a heat-map. The heat-map was then plotted on the covariance plane within the three-dimensional space of thigh, shank, and foot.

Kinematic data comparisons were done by using a paired Wilcoxon signed rank test to compare between before and after HAL therapy for each of acute and chronic groups, and by using an unpaired Wilcoxon signed rank test to compare between the healthy and each of Acute-pre, Acute-post, Chronic-pre, and Chronic-post groups. Due to the sample size, a *post-hoc* power test was applied to all significant data (1,000 times replication). Significance was considered when a *p* < 0.05 accompanied by a power test >80% was found. Marginal significance was considered with *p* < 0.05 and power test >50% (Hoenig and Heisey, 2001). All statistical analysis was carried out by using custom made scripts on MATLAB [version 8.4.0.150421(R2014B)] and RStudio (version 1.0.136).

## Results

**Figure 1:**
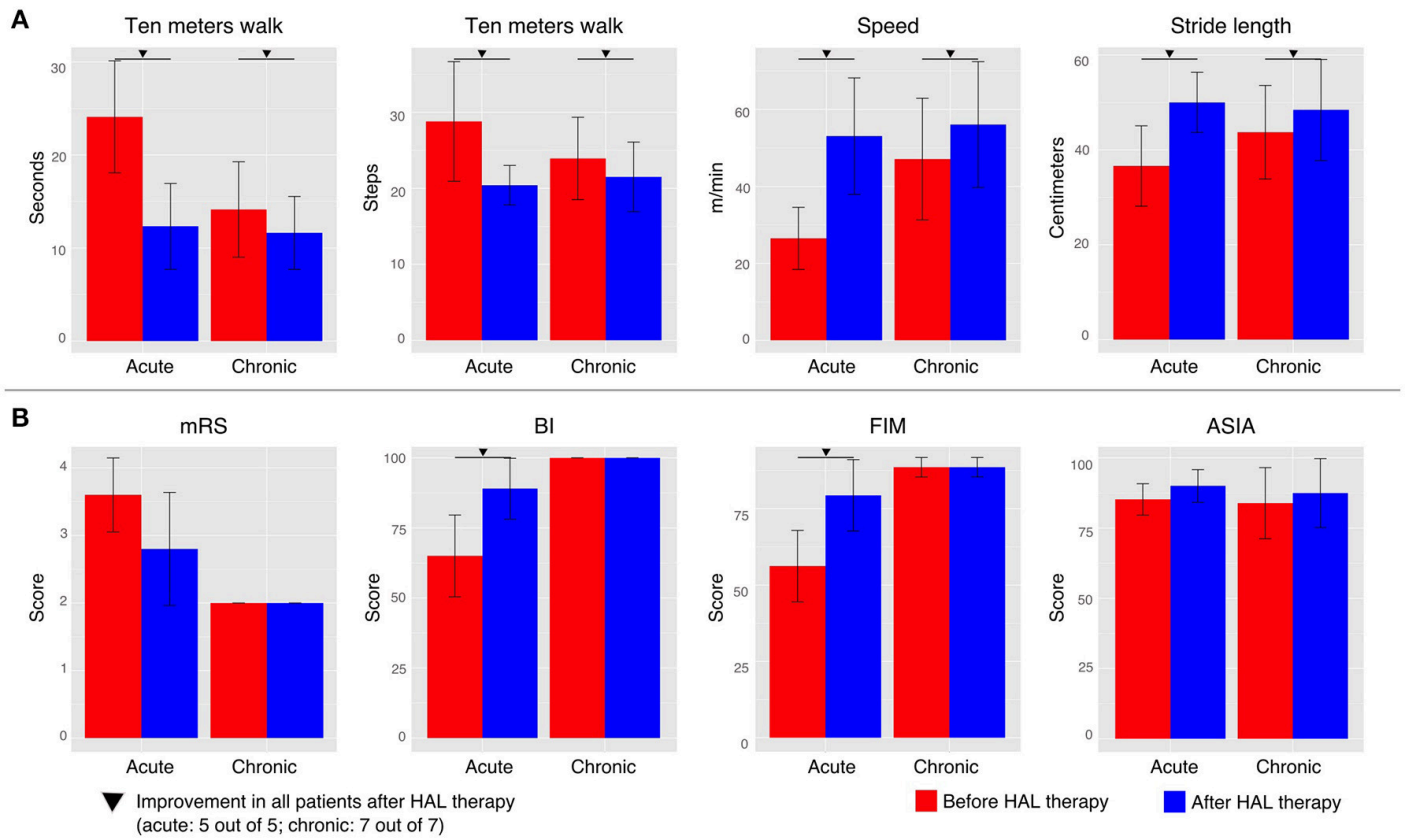
Walking performance and clinical evaluation. **(A)** The walking performance was assessed without fitting the robot during the 10 m walk test. **(B)** Clinical evaluation was performed by using the modified Ranking Scale (mRS), Barthel Index (BI), Functional Independence Measure (FIM, motor score), and the American Spinal Injury Association impairment scale (ASIA) in order to evaluate the degree of dependency of each patient. Patients were tested before the first HAL therapy and after the last one. Inverted triangle marks indicate improvement in all patients before and after HAL therapy (5 out to 5 for acute group and 7 out of 7 for chronic group).

In the present study, we found that HAL therapy improved the walking performance; the walking speed and stride length were increased, and the time and number of steps to cover 10 m were decreased, in all acute and chronic patients (Figure 1A). Positive effects were also found in the motor function scores; the BI and FIM scores were increased after HAL therapy for all acute patients (Figure 1B). mRS score was reduced in four, and stayed the same in one, of the acute patients. On the other hand, there was no change in the mRS, BI, or FIM functional evaluation scales of the chronic patients. ASIA scores did not show relevant changes for either group (Figure 1B). The improvements suggested possibility of beneficial effect of HAL for OPLL patients as previous studies (Aach et al., 2014; Fujii et al., 2016; Kasai and Takeda, 2016; Kubota et al., 2016). The effect of HAL therapy could be a reinforcement of the motor learning process during training, helping patients to reshape their motor function. Necessity for further investigation was considered.

**Figure 2:**
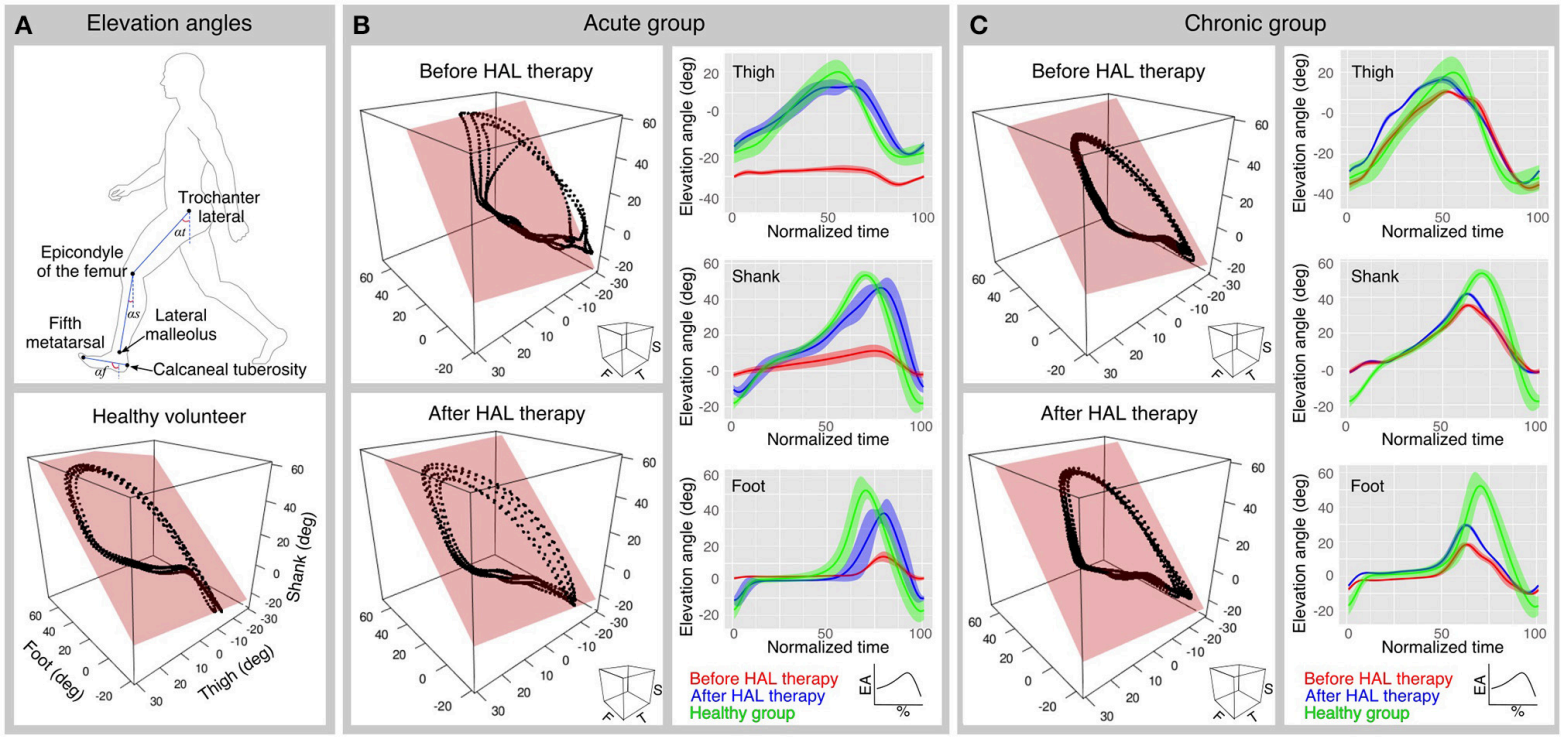
Planar covariation and peaks analysis. **(A)** Upper panel shows the segments used to calculate the elevation angles. Lower panel shows a planar covariation analysis plot from a healthy volunteer. **(B,C)** Planar covariation analysis sample data for one acute **(B)** and one chronic **(C)** patient before and after HAL therapy (left column). Each dotted trajectory corresponds to different strides of a single subject. Elevation angle profiles also were plotted before and after HAL therapy for each segment (right column); The normalized time corresponds to the percentage of the walking cycle; solid lines represent the average and the width of the highlighted area is given by the standard deviation.

**Figure 3:**
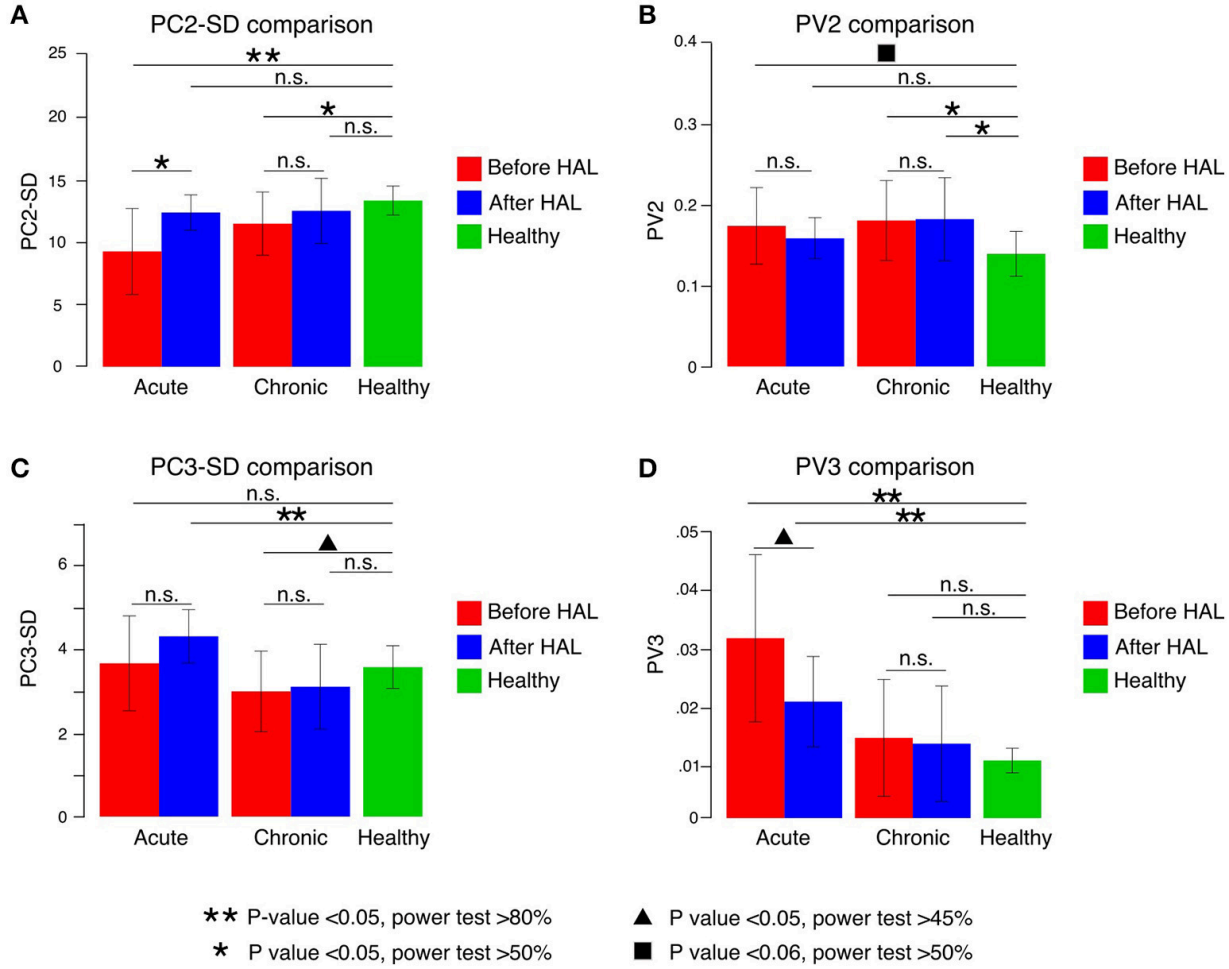
Principal components (PC) and percentage of variance (PV) comparisons. **(A)** PC2 SD increased significantly in acute patients after HAL therapy. Significant difference when compared to healthy volunteers was found in both groups before HAL therapy but not after HAL therapy. **(B)** PV2 comparisons before and after HAL therapy did not show significant changes in either group. When compared to healthy volunteers, acute patients showed a difference close to significance before HAL therapy only. Chronic group PV2 was significantly different from healthy before and after HAL therapy. **(C)** PC3-SD comparisons only showed significant difference from healthy group for acute patients after HAL therapy and chronic patients before HAL therapy. **(D)** PV3 comparisons showed a notable reduction after HAL therapy for acute group. Despite being closer to healthy group, it was still significantly different. Double asterisk marks refer to *P* < 0.05 and power test >80%; single asterisk notes *P* < 0.05 and power test >50%; triangle mark refers to *P* < 0.05 with power test >40%; square mark notes *P* < 0.06 and power test >50% and n.s. refers to non-significant changes.

In order to understand better the effect of HAL therapy, kinematics analysis was performed on the intersegmental correlation described by the covariation of thigh, shank, and foot EA (Figure 2A). At a glance, the shape of the loops described by patients were distorted in contrast with healthy volunteers evidencing impaired intersegmental coordination (Figures 2B–C). Comparison of PC2 standard deviation (PC2-SD) before and after HAL therapy was significantly different for acute group only (Figure 3A, PC2-SD mean; acute-pre: 9.30 ± 3.46, acute-post: 12.43 ± 1.41, chronic-pre: 11.54 ± 2.55, chronic-post: 12.57 ± 2.62. PC2-SD *P*-values; acute pre-post: < 0.01, power test: 64.4%, chronic pre-post: < 0.01, power test: 15.7%. Supplementary Tables 1, 2). The significant change of PC2-SD before and after HAL therapy for acute group seems to be beneficial regarding the results comparison contrasted to healthy volunteers; where PC2-SD increased for acute and chronic groups after HAL therapy, becoming closer to healthy participants (Figure 3A, PC2-SD mean; healthy: 13.40 ± 1.16. PC2-SD *P*-values; pre-acute vs. healthy: < 0.01, power test: 91%; post-acute vs. healthy: 0.039, power test: 39.8%; pre-chronic vs. healthy: 0.017, power test: 67.4%; post-chronic vs. healthy: 0.355. Supplementary Tables 1, 2). Comparison of PV2 before and after HAL therapy did not differ in acute or chronic group (Figure 3B, PV2 mean acute-pre: 0.17 ± 0.04, acute-post: 0.15 ± 0.02, chronic-pre: 0.18 ± 0.04, chronic-post: 0.18 ± 0.05. PV2 *P*-values; acute pre-post: 0.275, chronic pre-post: 0.808. Supplementary Tables 1, 2). Additionally, PV2 difference was found before but not after HAL therapy between acute patients and healthy volunteers (Figure 3B, PV2 mean; healthy: 0.14±0.03. PV2 *P*-values; pre-acute vs. healthy: 0.053, power test: 55.5%; post-acute vs. healthy: 0.139; pre-chronic vs. healthy: < 0.01, power test: 75.2%; post-chronic vs. healthy: < 0.01, power test: 76.3%. Supplementary Tables 1, 2). These results suggest that HAL therapy improved the actual width of the gait loop on the covariance plane for both acute and chronic groups, while improvement of proportional width of the loop was observed only for acute group (Figures 3A,B).

Deviation from covariation plane evaluated by PC3-SD comparison did not show significant difference between before and after HAL therapy for either of acute and chronic groups (Figure 3C, PC3-SD mean; acute-pre: 3.65 ± 1.13, acute-post: 4.30 ± 0.64, chronic-pre: 2.99 ± 0.96, chronic-post: 3.09 ± 1.01. PC3-SD *P*-values; acute pre-post: 0.083, chronic pre-post: 0.104. Supplementary Tables 1, 2). Comparison against healthy group showed a significant difference only for acute group after HAL. (Figure 3C, PC3-SD mean; healthy: 3.57 ± 0.51. PC3-SD *P*-values; pre-acute vs. healthy: 0.391, post-acute vs. healthy: < 0.01, power test: 82.5%; pre-chronic vs. healthy: 0.011, power test: 48.2%; post-chronic vs. healthy: 0.042, power test: 30.2%. Supplementary Tables 1, 2). However, proportional deviation from covariation plane evaluated by PV3 demonstrated a trend close to significance showing a better plane fitting after HAL therapy for acute but not for chronic group; quantitative comparison between PV3 before and after HAL therapy were close to significance for acute group only (Figure 3D, PV3 mean acute-pre: 0.03 ± 0.01, acute-post: 0.02 ± 0.007, chronic-pre: 0.013 ± 0.009, chronic-post: 0.012 ± 0.009. PV3 *P*-values; acute pre-post: 0.027, power test: 47.2%, chronic pre-post: 0.295. Supplementary Tables 1, 2). Comparisons against healthy volunteers showed significant difference of PV3 for acute patients before and after HAL therapy but not for chronic patients (Figure 3D, PV3 mean; healthy: 0.009 ± 0.002. PV3 *P*-values; pre-acute vs. healthy: < 0.01, power test: 96.5%; post-acute vs. healthy: < 0.01, power test: 94.6%; pre-chronic vs. healthy: 0.473; post-chronic vs. healthy: 0.447. Supplementary Tables 1, 2). Tendency of PV3 recovery was observed for acute group suggesting a positive change in planarity of coordination after HAL therapy (Figure 3D), although it did not reach a level comparable to healthy volunteers. On the other hand, comparisons between chronic and healthy groups did not show significant difference.

Plane orientation evaluated by the NV difference between acute and chronic groups was also significantly different (acute angle mean: 6.82 ± 4.93 deg, chronic angle mean: 2.58 ± 2.18 deg. Acute-chronic *P*-value 0.026, power test 63.1%) suggesting that plane orientation changes after HAL therapy were larger for acute group than the changes found for chronic group. The angular deviation of U3 vector from averaged healthy volunteers changed mainly for acute group only (U3 mean. Acute-pre: 7.720 ± 0.56 deg, acute-post: 8.168 ± 0.19 deg. *P*-value acute pre-post < 0.01, power test 54.9%. Chronic-pre: 8.085 ± 0.23 deg, chronic-post: 8.1366 ± 0.278 deg. *P*-value chronic pre-post: 0.049, power test: 6.4%).

Peak comparisons before and after HAL therapy (max peak, min peak, and max-min difference) were used to evaluate the limb movement range during gait. For acute group, foot EA comparisons were significantly different for the max peak and max-min difference (max foot mean; pre: 34.01 ± 16.88 deg, post: 55.27 ± 7.44 deg, pre-post *P*-value < 0.01, power test: 81.5%. min foot mean; pre: −15.48 ± 8.84 deg, post: 24.88 ± 4.58 deg, pre-post *P*-value < 0.01, power test: 72%; max-min difference foot mean; pre: 49.49 ± 23.8 deg, post: 80.16 ± 9.50 deg, pre-post *P*-value < 0.01, power test: 85.4%) suggesting improvement of the foot excursion. Other peaks did not show significance (*P*-values for max thigh: 0.275; max shank: 0.037, power test: 42.3%; min thigh: 0.492; min shank 0.019, power test 38.1%; max-min diff thigh: 0.083; max-min diff shank: < 0.01, power test: 52.4%). On the other hand, chronic group patients did not show significant changes among peaks (*P*-values for max thigh: 0.761; max shank: 0.104; max foot: < 0.01, power test: 23.7%; min thigh: 0.135; min shank: 0.583; min foot: 0.808; max-min diff thigh: < 0.01, power test: 49.2%; max-min diff shank: < 0.01, power test: 12.8%; max-min diff foot: < 0.01, power test: 19.4%) (Supplementary Tables 3, 4). This finding may suggest that acute patients foot coordination improved giving the patients a longer stride and better foot clearance. Although shank EA peaks did not show significance for chronic patients, its difference increased after HAL therapy (shank EA difference: pre: 54.313 deg, post: 57.518 deg) (Figures 2B,C, right column); this increment may be related to an enlargement of movement range, and may be the reason of the larger stride length and improved limb excursion after HAL therapy in chronic patients.

**Figure 4:**
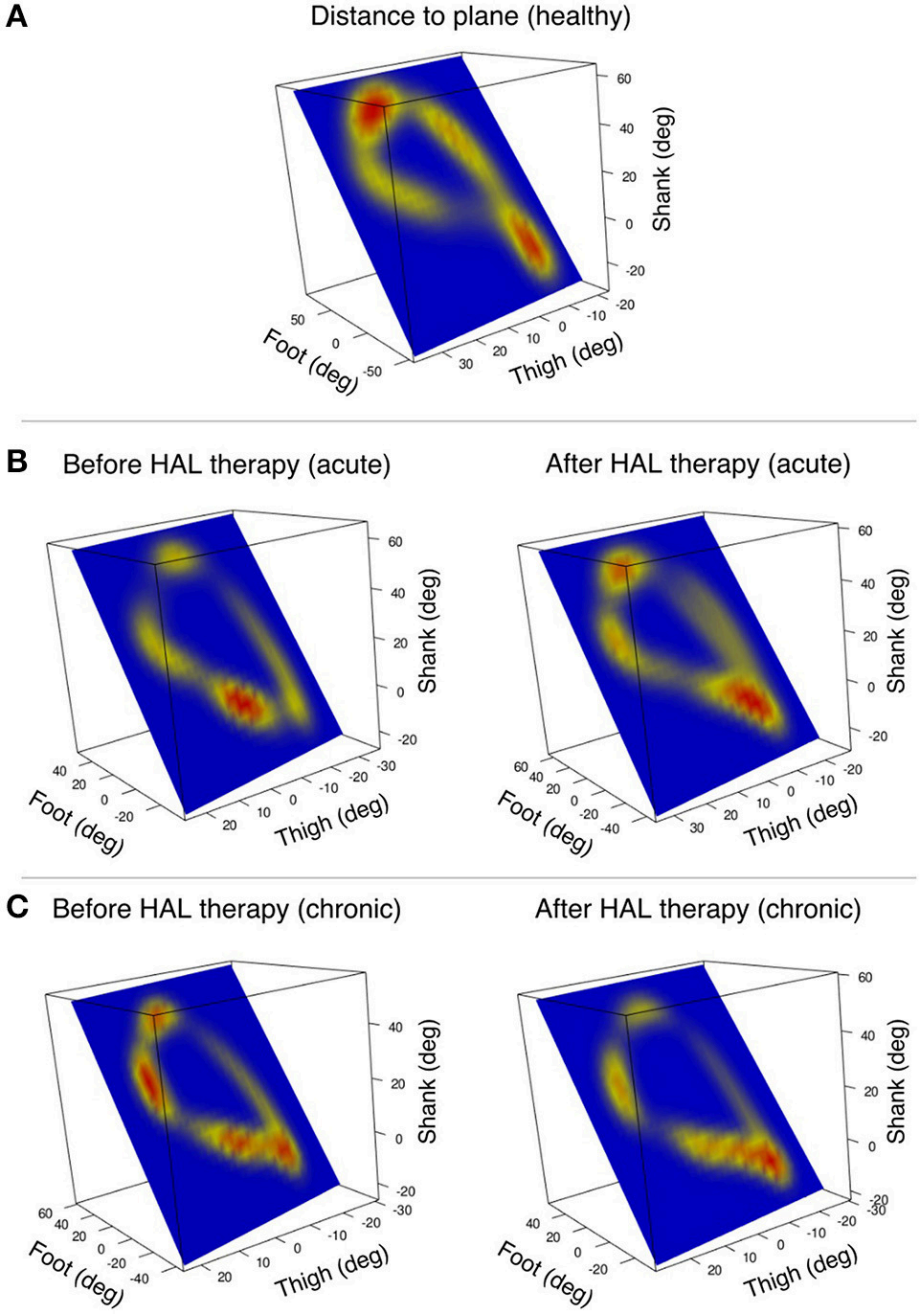
Pattern of deviation from the covariance plane through gait cycle is visualized by heat-maps. The heat-maps are plotted on the covariance plane within the tridimensional space of thigh, shank, and foot. **(A)** An example of a healthy volunteer shows deviation from the plane mainly in the zones corresponding to heel strike and toe off. **(B)** Acute patient's deviation from the plane was shifted before HAL therapy, but the pattern was recovered after HAL therapy (one patient single leg example). **(C)** Chronic patients had several hot spots marking deviation from the plane that changed briefly after HAL therapy (one patient single leg example).

Heat maps were used to assess the pattern of deviation from the covariance plane through the gait cycle. The pattern found in healthy volunteers showed hot spots in the areas related to heel strike and toe off (Figure 4A). However, patients from the acute group tended to have shifted hot spots which tended to recover the healthy pattern after HAL therapy (Figure 4B). Chronic patients had hot spots sprayed around the gait loop; after HAL therapy, there was a tendency to reduce these hot spots (Figure 4C). The resemblance of acute pattern after HAL therapy to healthy volunteers suggests an improvement in limb motion and excursion. This data redistribution support our theory about coordination improvement after HAL therapy for acute OPLL patients.

## Discussion

It has been well-established that covariation of the EA corresponding to the lower limbs (thigh, shank, and foot) is consistent across subjects during bipedal walking in normal conditions (Borghese et al., 1996; Bianchi et al., 1998a; Ivanenko et al., 2008), and it is preserved during different gait perturbations (Bianchi et al., 1998b; Grasso et al., 1998; Ivanenko et al., 2002; Noble and Prentice, 2008). Also, there are previous studies of planar covariation in different medical conditions as stroke (Bleyenheuft et al., 2009; Chow and Stokic, 2015), lower limb amputations (Leurs et al., 2012), and cerebellar ataxia (Martino et al., 2014) where the intersegmental coordination is maintained independently of the medical condition affecting the gait pattern. However, planar covariation is preserved only in amputees when using a prosthesis independently of the adaptation time (Leurs et al., 2012). Patients with impairment of the central motor pathways often show alterations in planar covariation of the EA suggesting that it has a central origin (Ivanenko et al., 2008; Bleyenheuft et al., 2009; Martino et al., 2014; Chow and Stokic, 2015).

It is said that the central nervous system may use the intersegmental coordination as a strategy to simplify the gait control by reducing the effective degrees of freedom of muscle activation (Lacquaniti et al., 2002). Additionally, studies in toddlers have shown that the planar covariation patterns evidenced in adults starts to appear when mature gait patterns are achieved, suggesting that a coordinated behavior is controlled centrally instead of pure biomechanical constraints (Cheron et al., 2001). This observation reinforces the statement that planar covariation alterations have a central component and does not depend on a simple biomechanical effect. Therefore, the alteration of the gait coordination may suggest the central nervous system recurring to alternative strategies to provide functional motor output.

Our kinematic analysis showed that the actual width of covariation loop was broadened for both acute and chronic patients after HAL therapy (Figure 3A). The widths became closer to healthy controls, suggesting improvement. The proportional width and planarity were improved for acute but not for chronic patients (Figures 3B,D). These improvements of planar covariation suggest a beneficial effect of HAL at the level of the central nervous system, including the spinal cord and the brain especially for acute patients, the changes leading them to functional recovery of gait. In acute patients, where myelopathy started recently and poor posture due to motor disturbances may have not been established yet, the conditions to get a marked improvement were better than for chronic patients. Thus, acute patients got improvement in their walking performance and coordination after HAL therapy probably secondary to spinal remodeling and reinforcement of central pattern generators. Also, the improvement of movement range evaluation related to foot peaks suggested that HAL treatment improved central coordination rather than segment-wise adaptation. For chronic patients, where myelopathy had become a chronic condition and the patient might have developed non-ergonomic postures to achieve a functional gait, the effect of HAL therapy was evident for walking performance but subtle for coordination related to planar covariation. We believe that chronic patients had earned already a certain degree of coordination that allowed them to perform basic daily life activities before HAL therapy. Still, the walking performance was improved for them after HAL therapy. Therefore, we may infer that HAL therapy helped them to adapt their strategies of coordinated limb motion control to improve the speed and stride length.

Previous studies have shown the beneficial effects of exercising as a non-invasive treatment to provide rhythmic stimulation to the spinal cord (Sandrow-Feinberg and Houlé, 2015; Gad et al., 2017). Stimulation of spinal pathways in a rhythmic fashion may provide a beneficial effect in gait recovery by enhancing the afferent input to the spinal cord and activating the central pattern generators. This activity also increase the central nervous system plasticity, neurogenesis, and remodeling (Dunlop, 2008; Houle and Côté, 2013); however, in patients with motor disabilities regular exercise generally is not an option. Rehabilitation programs are expensive, demanding and generally cannot be offered for long periods. Additionally, post-surgical patients do not have immediate access to an exercise source during the hospitalization term. The robot suit HAL treatment offers a unique opportunity because of its ability of supplying a constant feedback from the patient's own bioelectrical signals, providing support in accordance with the patient's voluntary gait during training. We believe that the HAL's feedback exerts a direct effect on the reshaping process of muscular recruitment, accelerating the gait restructuration to generate an improved walking pattern. We also think that an intense workout program as designed for HAL therapy may induce plasticity in the spinal cord and cortex leading to neurogenesis and reorganization of the available pathways to improve motor performance. Acute patients after a recent lesion may achieve faster improvement after HAL therapy by the degree of plasticity and rewiring process in the central nervous system. On the other hand for chronic patients, there was no marked effect in coordination. However, the walking performance of these patients were improved after HAL therapy suggesting that, although plasticity and remodeling process at the spinal cord level may be slower than acute patients, a lower level of neuroplasticity is still able to induce detectable changes during voluntary gait of chronic patients.

Additionally, the coincident improvement of the clinical evaluation of functional scores (Figure 1B) and the recovery of the planar covariation for acute patients (Figure 3) may suggest an association between the changes in the walking performance and gait coordination. We consider that gait coordination analysis can be applied as an objective functional evaluation of a patient's progress during rehabilitation program.

Apart from HAL, there are other robotic assisted therapies available for gait rehabilitation. The end effector-type robotic device known as “Gait Trainer” (Werner et al., 2002; Tong et al., 2006; Peurala et al., 2009) and the exoskeleton type known as Lokomat (Mayr et al., 2007) has been used for stroke patients. The subjects showed improvement in gait performance after the intervention when evaluating gait parameters and clinical scores. The Lokomat has been used also for spinal cord injury, where beneficial effects were found in clinical scores and gait parameters (Labruyere and Van Hedel, 2014; Nam et al., 2017). Some studies have used robotic assisted therapy combined with additional treatments, also finding a beneficial effect in the patients' clinical scores (Schwartz et al., 2009; Bae et al., 2014). In contrast to these robots, HAL provides motion assistance during gait based on detected bioelectric signals of the peripheral neuro-muscular activity relevant to the lower limb joint motions, helping the user to perform intended voluntary motion in real time. This feedback is considered to compensate for the disordered loop of active motion planning, execution and sensation. We hypothesize that this function of HAL contributes to reshaping of gait and neural systems behind locomotion, as described by the gait coordination changes shown in this study. To our knowledge, this is the first report regarding analysis on gait coordination of patients before and after robotic assisted rehabilitation.

This study has limitations regarding the size of the population. Also, it was not possible to compare our results to OPLL patients that did not receive HAL therapy to discard the possibility of recovery independent of the intervention. Although data cannot be extrapolated to all the population, this study give the first insight regarding robotic rehabilitation and changes of gait coordination analyzed by planar covariation. Further studies with larger populations should be done to continue exploring the effect of HAL therapy on gait coordination.

## Author contributions

SP and HK collected, analyzed, and interpreted the data; wrote and drafted the manuscript. SK recruited the patients and administered HAL therapy. TA operated all the patients from acute group. YuS supported HAL therapy. AM provided important comments for the clinical part of the study and helped developing of HAL therapy. YoS originally developed the robot suit HAL and conceived the idea of HAL therapy. MY operated all patients from chronic group, and developed HAL therapy for OPLL patients. KS designed the analysis and provided essential insight for the paper. All authors made critical revisions of the manuscript and approved the final version.

### Conflict of interest statement

YoS is the C.E.O., shareholder, and director of CYBERDYNE Inc. which produces the robot suit HAL. CYBERDYNE was not involved in study design, data collection, analysis, writing, or submission of this article. The other authors declare that the research was conducted in the absence of any commercial or financial relationships that could be construed as a potential conflict of interest.
