# Supplementary material for: Reshaping of Gait Coordination by Robotic Intervention in Myelopathy Patients After Surgery
Source: Front Neurosci. 2018 Mar 2;12:99. doi: 10.3389/fnins.2018.00099 (PMC5840280; doi:10.3389/fnins.2018.00099)
Supplement: Supplementary file 1 [file DataSheet1.pdf]

Supplementary Material

Reshaping of Gait Coordination by Robotic Intervention in Myelopathy Patients after Surgery

Sandra Puentes*, Hideki Kadone, Shigeki Kubota, Tetsuya Abe, Yukiyo Shimizu, Aiki Marushima, Yoshiyuki Sankai, Masashi Yamazaki, Kenji Suzuki.

***Correspondence:** Sandra Puentes: sandra@ccr.tsukuba.ac.jp

Stage	Data	Mean pre-HAL	Mean post-HAL
**Acute**	PC2 SD	9.30±3.46	12.43±1.41
**Acute**	PV2	0.17±0.04	0.15±0.02
**Chronic**	PC2 SD	11.54±2.55	12.57±2.62
**Chronic**	PV2	0.18±0.04	0.18±0.05
**Acute**	PC3SD	3.65±1.13	4.30±0.64
**Acute**	PV3	0.03±0.01	0.02±0.007
**Chronic**	PC3SD	2.99±0.96	3.09±1.01
**Chronic**	PV3	0.013±0.009	0.012±0.009
**Healthy**	PC2SD	13.40±1.16	-
**Healthy**	PV2	0.14±0.03	-
**Healthy**	PC3SD	3.57±0.51	-
**Healthy**	PV3	0.009±0.002	-

Supplementary Table 1. Average data from PCA data before and after HAL treatment

Stage	Data	Comparison	P-value	Observed power (%)
**Acute**	PC2 SD	Pre Vs Post	**<0.01**	**64.4**
**Acute**	PV2	Pre Vs Post	0.275	13.3
**Chronic**	PC2 SD	Pre Vs Post	<0.01	15.7
**Chronic**	PV2	Pre Vs Post	0.808	3.9
**Acute**	PC3SD	Pre Vs Post	0.083	27.5
**Acute**	PV3	Pre Vs Post	0.027	47.2
**Chronic**	PC3SD	Pre Vs Post	0.104	14.9
**Chronic**	PV3	Pre Vs Post	0.295	5.9
**Mix**	PC2SD	Pre.ac Vs H	**<0.01**	**91.0**
**Mix**	PC2SD	Post.ac Vs H	0.039	39.8
**Mix**	PC2SD	Pre.ch Vs H	**0.017**	**67.4**
**Mix**	PC2SD	Post.ch Vs H	0.355	18.2
**Mix**	PV2	Pre.ac Vs H	**0.053**	**55.5**
**Mix**	PV2	Post.ac Vs H	0.139	38.4
**Mix**	PV2	Pre.ch Vs H	**<0.01**	**75.2**

**Mix**	PV2	Post.ch Vs H	**<0.01**	**76.3**
**Mix**	PC3SD	Pre.ac Vs H	0.391	9.9
**Mix**	PC3SD	Post.ac Vs H	**<0.01**	**82.5**
**Mix**	PC3SD	Pre.ch Vs H	0.011	48.2
**Mix**	PC3SD	Post.ch Vs H	0.042	30.2
**Mix**	PV3	Pre.ac Vs H	**<0.01**	**96.5**
**Mix**	PV3	Post.ac Vs H	**<0.01**	**94.6**
**Mix**	PV3	Pre.ch Vs H	0.473	31.6
**Mix**	PV3	Post.ch Vs H	0.447	21.1

Supplementary Table 2: Statistical comparison using Wilcoxon signed rank test (paired) and post hoc power tests. Observed power from 50-79% was considered **marginally significant**. Observed power above 89% was considered **significant** (pre: before HAL therapy, Post: after HAL therapy, ac: acute, ch: chronic, H: healthy).

Stage	Data	Mean pre-HAL (deg)	Mean post-HAL (deg)
**Acute**	Max thigh	2.41±26.3	19.99±3.87
**Acute**	Min thigh	-26.97±11.4	-20.42±4.80
**Acute**	Diff thigh	29.38±18.7	40.42±6.81
**Acute**	Max shank	33.69±18.2	50.15±4.22
**Acute**	Min shank	-9.83±4.85	-14.90±4.67
**Acute**	Diff shank	43.52±21.6	65.05±6.22
**Acute**	Max foot	34.01±16.88	55.27±7.44
**Acute**	Min foot	-15.48±8.84	-24.88±4.58
**Acute**	Diff foot	49.49±23.8	80.16±9.50
**Chronic**	Max thigh	29.88±73.7	14.71±3.26
**Chronic**	Min thigh	-5.07±73.7	-23.89±5.39
**Chronic**	Diff thigh	34.96±4.58	38.61±5.58
**Chronic**	Max shank	46.43±7.98	48.86±5.75
**Chronic**	Min shank	-7.87±6.65	-8.65±5.68
**Chronic**	Diff shank	54.31±10.3	57.51±8.37
**Chronic**	Max foot	49.05±13.2	55.71±10.9
**Chronic**	Min foot	-14.53±8.37	-14.62±6.61
**Chronic**	Diff foot	63.59±14.3	70.34±11.5
**Healthy**	Max thigh	19.88±5.46	-
**Healthy**	Min thigh	-21.82±5.48	-
**Healthy**	Diff thigh	41.71±4.51	-
**Healthy**	Max shank	54.56±2.56	-
**Healthy**	Min shank	-19.75±2.99	-
**Healthy**	Diff shank	74.31±4.33	-
**Healthy**	Max foot	67.08±5.64	-
**Healthy**	Min foot	-26.65±7.06	-
**Healthy**	Diff foot	93.73±10.1	-

Supplementary Table 3: Average data from peak analysis of thigh, shank and foot elevation angles before and after HAL therapy

Stage	Data	Comparison	P-value	Observed power (%)
**Acute**	Max thigh	Pre Vs Post	0.275	23.3
**Acute**	Max shank	Pre Vs Post	0.037	42.3
**Acute**	Max foot	Pre Vs Post	**<0.01**	**81.5**
**Acute**	Min thigh	Pre Vs Post	0.492	8.7
**Acute**	Min shank	Pre Vs Post	0.019	38.1
**Acute**	Min foot	Pre Vs Post	**<0.01**	**72.0**
**Acute**	Diff thigh	Pre Vs Post	0.084	19.5
**Acute**	Diff shank	Pre Vs Post	<0.01	52.4
**Acute**	Diff foot	Pre Vs Post	**<0.01**	**85.4**
**Mix**	Max thigh	Pre.ac Vs H	<0.01	28.2
**Mix**	Max shank	Pre.ac Vs H	**<0.01**	**69.8**
**Mix**	Max foot	Pre.ac Vs H	**<0.01**	**99.9**
**Mix**	Min thigh	Pre.ac Vs H	<0.01	6.3
**Mix**	Min shank	Pre.ac Vs H	**<0.01**	**98.6**
**Mix**	Min foot	Pre.ac Vs H	**<0.01**	**76.4**
**Mix**	Diff thigh	Pre.ac Vs H	<0.01	27.4
**Mix**	Diff shank	Pre.ac Vs H	**<0.01**	**86.2**
**Mix**	Diff foot	Pre.ac Vs H	**<0.01**	**99.5**
**Mix**	Max thigh	Post.ac Vs H	1	25
**Mix**	Max shank	Post.ac Vs H	**<0.01**	**65.3**
**Mix**	Max foot	Post.ac Vs H	**<0.01**	**97.1**
**Mix**	Min thigh	Post.ac Vs H	0.938	5.6
**Mix**	Min shank	Post.ac Vs H	**0.011**	**64.6**
**Mix**	Min foot	Post.ac Vs H	0.659	7.1
**Mix**	Diff thigh	Post.ac Vs H	0.659	5.6
**Mix**	Diff shank	Post.ac Vs H	**<0.01**	**91.0**
**Mix**	Diff foot	Post.ac Vs H	**<0.01**	**81.8**
**Chronic**	Max thigh	Pre Vs Post	0.761	14.3
**Chronic**	Max shank	Pre Vs Post	0.104	11.0
**Chronic**	Max foot	Pre Vs Post	<0.01	23.7
**Chronic**	Min thigh	Pre Vs Post	0.135	19.9
**Chronic**	Min shank	Pre Vs Post	0.583	6.6
**Chronic**	Min foot	Pre Vs Post	0.807	5.4
**Chronic**	Diff thigh	Pre Vs Post	<0.01	49.2
**Chronic**	Diff shank	Pre Vs Post	<0.01	12.8
**Chronic**	Diff foot	Pre Vs Post	<0.01	19.4
**Mix**	Max thigh	Pre.ch Vs H	0.047	13.5
**Mix**	Max shank	Pre.ch Vs H	**0.017**	**69.8**
**Mix**	Max foot	Pre.ch Vs H	**<0.01**	**86.2**
**Mix**	Min thigh	Pre.ch Vs H	0.886	16.1
**Mix**	Min shank	Pre.ch Vs H	**<0.01**	**98.4**
**Mix**	Min foot	Pre.ch Vs H	**<0.01**	**89.8**
**Mix**	Diff thigh	Pre.ch Vs H	**<0.01**	**75.6**
**Mix**	Diff shank	Pre.ch Vs H	**<0.01**	**99.2**
**Mix**	Diff foot	Pre.ch Vs H	**<0.01**	**99.7**
**Mix**	Max thigh	Post.ch Vs H	**<0.01**	**62.8**
**Mix**	Max shank	Post.ch Vs H	**<0.01**	**67.9**
**Mix**	Max foot	Post.ch Vs H	**<0.01**	**59.7**

**Mix**	Min thigh	Post.ch Vs H	0.142	20.6
**Mix**	Min shank	Post.ch Vs H	**<0.01**	**98.5**
**Mix**	Min foot	Post.ch Vs H	**<0.01**	**93.9**
**Mix**	Diff thigh	Post.ch Vs H	0.313	10.3
**Mix**	Diff shank	Post.ch Vs H	**<0.01**	**99.4**
**Mix**	Diff foot	Post.ch Vs H	**<0.01**	**97.0**

Supplementary Table 4: Peak analysis statistical comparison using Wilcoxon signed rank test (paired) and post hoc power tests. Observed power from 50-79% was considered marginally significant. Observed power above 89% was considered significant (pre: before HAL therapy, Post: after HAL therapy, ac: acute, ch: chronic, H: healthy, diff: max-min peaks difference).
